# Effect of Inorganic Salts on Synthesis of Poly(glycidyl methacrylate) Microspheres, Their Functionalization with Poly(ethyleneimine) and Evaluation of Its Use for Removal of Acid Red 27, Acetaminophen and Nitrites

**DOI:** 10.3390/polym18070835

**Published:** 2026-03-29

**Authors:** Jina M. Martínez, Marisol Rincón, Manuel Palencia

**Affiliations:** 1 Reseach Group in Science with Technological Application (GI-CAT), Department of Chemistry, Faculty of Natural and Exact Sciences, Universidad del Valle, Cali 760032, Colombia; jina.martinez@correounivalle.edu.co (J.M.M.); marisol.rincon@correounivalle.edu.co (M.R.); 2 Mindtech Research Group (Mindtech-RG), Mindtech S.A.S., Montería 230002, Colombia

**Keywords:** poly(glycidyl methacrylate), suspension polymerization, polyethyleneimine, anionic dyes, nitrites, emerging pharmaceutical pollutant

## Abstract

Poly(glycidyl methacrylate) (PGMA) is a polymer containing epoxy groups in its side chains, which makes it a suitable platform for the development of functional materials. In this study, crosslinked PGMA-based microspheres were synthesized by suspension polymerization using N,N′-methylenebisacrylamide as a crosslinker, and the effect of incorporating inorganic additives (InAds) (NaCl, CaCO_3_, and MgO nanoparticles) during synthesis was evaluated. In all cases, solid microspheres were obtained, exhibiting variations in particle size, sphericity, and aggregation depending on the type and amount of InAds. Thermal stability was characteristic of crosslinked PGMA (i.e., a single broad thermal transition in ~80–110 °C), while water absorption remained within a narrow range (80–120% for t = 40 min). In addition, the number of epoxy groups per gram of sample was 4.83 ± 0.02 mmol g^−1^. Selected microspheres were subsequently functionalized with polyethyleneimine (PEI) to obtain graft polymers (PGMA–PEI) and evaluated for the adsorption of three model contaminants: Acid Red 27 (AR-27), nitrites, and acetaminophen. PGMA–PEI showed high affinity toward AR-27 and nitrites, achieving high removal efficiencies at acidic and neutral pH, with rapid adsorption kinetics consistent with a pseudo-second-order model, attributed to electrostatic interactions between protonated amine groups and anions. At pH 11, anion desorption was promoted, enabling partial material regeneration. The results highlight the potential of PGMA–PEI microspheres for the removal of AR-27 (maximum retention ~0.25 mg of dye/g of polymer) and nitrites (maximum retention ~0.023 mg of ${NO}_{2}^{-}$/g of polymer), whereas acetaminophen removal was not evidenced.

## 1. Introduction

Poly(glycidyl methacrylate) (PGMA) is a functional polymer derived from glycidyl methacrylate (GMA). In addition, it is of significant importance in materials science due to its ease of chemical modification through post-synthesis reactions, resulting from the presence of highly labile epoxy groups. These groups can undergo ring-opening reactions with a wide variety of nucleophiles, including amines, thiols, alcohols, and carboxylic acids [1,2,3]. The intrinsic reactivity of the epoxy groups, together with the favorable mechanical and thermal properties of PGMA, makes it a material of choice for a wide range of applications, from biotechnology to environmental remediation [4]. PGMA can be synthesized using various polymerization techniques, with free-radical polymerization being usually employed due to its simplicity and effectiveness [5]. Among the available methodologies, suspension polymerization has attracted particular attention owing to its ability to produce polymeric microspheres with controllable size distributions, scalability, and desirable morphological properties [6,7].

**Figure 1:**
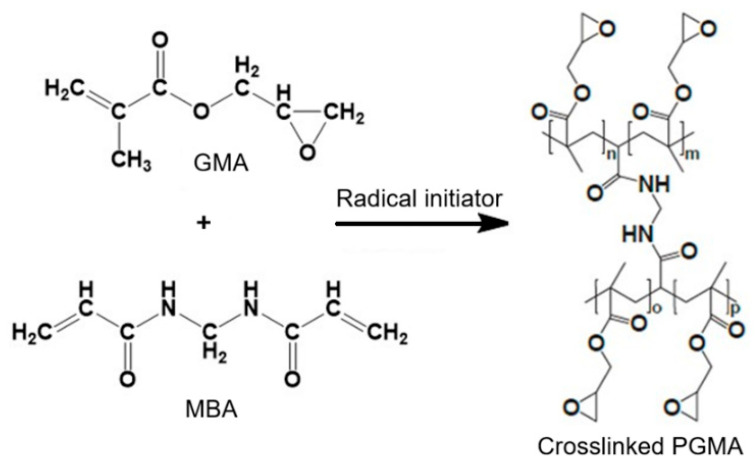
Illustration of free radical polymerization of GMA in the presence of MBAAm as a crosslinking agent.

In such systems, the addition of a crosslinking agent is critical for defining the internal structure of the particles and their mechanical and chemical stability. In particular, N,N′-methylenebisacrylamide (MBAAm) is a hydrophilic crosslinker frequently employed for its ability to generate compact three-dimensional networks. During polymerization, MBAAm reacts to link PGMA chains, forming a denser and more stable network (see Figure 1), which significantly enhances the mechanical strength of the particles as well as their stability in liquid media. This, in turn, allows control over the swelling degree of the material, a feature that is particularly important in applications involving the retention or release of compounds in aqueous solutions [8,9]. Likewise, the composition of the aqueous phase in suspension systems can have a decisive effect on the formation and characteristics of the microspheres. The addition of inorganic salts can modify viscosity, interfacial tension, and emulsion stability, thereby directly influencing the size, morphology, and size distribution of the resulting microspheres [10,11]. Understanding these variables is essential for optimizing the design and synthesis of new and advanced functional materials.

On the other hand, considering the advantages of PGMA resins, once synthesized, these microspheres can be further functionalized due to the reactivity of their epoxy groups. This functionalization has been widely exploited to introduce chelating, ionic, catalytic, or bioactive groups onto the polymer surface, thereby enhancing performance in specific applications. For example, the modification of PGMA with polymers such as polyethyleneimine (PEI) has been shown to be an effective strategy for incorporating amino groups on the material surface [12].

In this context, the present work focused on the synthesis of PGMA resins via suspension polymerization, primarily evaluating the impact of different salts in the PVA aqueous phase on the formation and morphological properties of the resulting microspheres. Subsequently, taking advantage of the reactivity of the epoxy groups present in the PGMA resins, chemical modification with PEI was carried out to introduce functional amino groups. Finally, the modified material was evaluated as an adsorbent for the removal of different compounds from aqueous systems: (i) acetaminophen (ACT) which is a globally marketed, over-the-counter, and widely used drug [13,14]; (ii) Acid Red 27 (AR-27) which is a synthetic azo and anionic dye used in the textile, paper, leather and coatings industries, it is important to clarify that it is not used in food applications, and given its azo nature it can be persistent in the environment and potentially toxic [15,16,17], and (iii) nitrite ions (${NO}_{2}^{-}$) whose removal is of great importance in aquaculture since this oxoanion is highly toxic to fish and other aquatic organisms even at relatively low concentrations, so its presence and accumulation compromise both animal health and the productive viability of fish farming systems [18,19,20].

ACT removal from aqueous solutions has been achieved through several methods, including adsorption onto sludge (with removal efficiencies ranging from 14.3 to 100%), as well as biodegradation techniques, which have typically been investigated in laboratory-scale studies using membrane bioreactors. However, these systems require precise temperature control (e.g., 17 ± 1 °C) and operate at concentrations around 3 µg/L under both aerobic and anaerobic conditions [21]. Reverse osmosis has also been applied; however, it is characterized by high energy consumption and is therefore not considered suitable for agricultural or, in many cases, for municipal wastewater treatment plants [22,23]. In contrast, the range of methods available for the removal of synthetic dyes is considerably broader. Common approaches include flocculation using adsorbents such as biochar, activated carbon, fibers, and polymers, among others [24], as well as photodegradation processes (e.g., UV/H_2_O_2_, UV/TiO_2_), electrocoagulation, and chemical oxidation methods (e.g., chlorination and ozonation) [25].

On the other hand, nitrite accumulation results from the interplay of multiple factors in aquaculture production systems. Among the most relevant are pH, temperature, low dissolved C/N ratios, high nitrate concentrations, and elevated dissolved oxygen levels, among others. A key characteristic that distinguishes nitrite from the other two compounds of interest in this study is that nitrite adsorption using, by instance, activated carbon, is generally inefficient, and, consequently, a limited removal has been reported via precipitation [26]. Reported nitrite removal methods include chemical and catalytic reduction, ion exchange, reverse osmosis, and electrodialysis [27]. Additional approaches for nitrite removal include electrical stimulation, which is particularly effective under low C/N ratios and high nitrate concentrations, supramolecular adsorbent-based systems, and denitrifying anaerobic methane oxidation [26,28].

**Figure 2:**
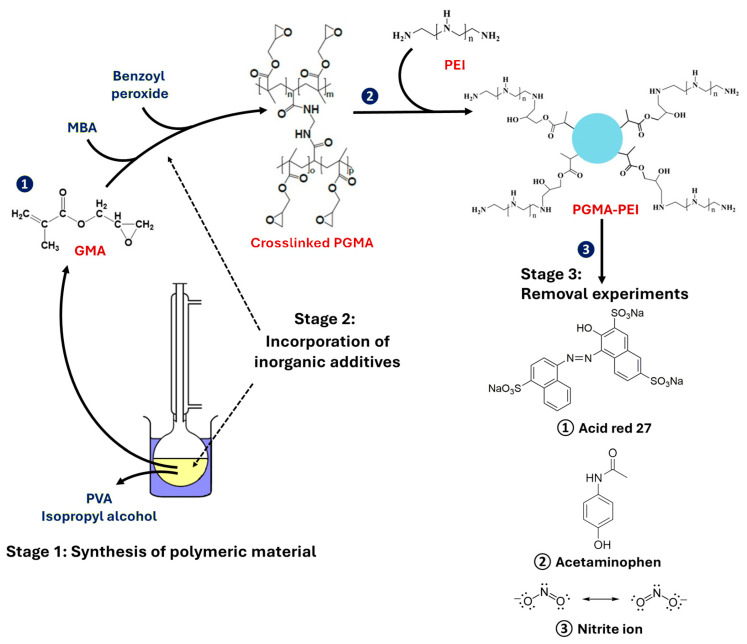
Description of study: Stage 1 (synthesis of PGMA), Stage 2 (study of the effect of incorporating inorganic additives on polymer) and Stage 3 (removal experiments of pollutants: acid red, acetaminophen and nitrite ion in aqueous solution).

This research had three objectives: (i) Studying the effect of incorporating inorganic additives (InAds) into the aqueous phase of suspension polymerization of PGMA; (2) obtaining PGMA microspheres grafted with PEI (PGMA-PEI); and (3) evaluating the removal of contaminants of environmental interest in aqueous solution (i.e., ACT, AR-27 and ${NO}_{2}^{-}$) using PGMA-PEI (see Figure 2).

## 2. Materials and Methods

### 2.1. Reagents

Magnesium hydroxide (Mg(OH)_2_, Merck, Rahway, NJ, USA), sodium carbonate (Na_2_CO_3_, Merck, Rahway, NJ, USA), and calcium chloride (CaCl_2_, Sigma-Aldrich, St. Louis, MO, USA) were used as precursors for calcium carbonate nanoparticles (CaCO_3_NPs) and magnesium oxide nanoparticles (MgONPs). Sodium hydroxide (NaOH, 98%, Sigma-Aldrich, USA) was used as an auxiliary reagent in the synthesis of the CaCO_3_NPs. Sodium chloride (NaCl, 99.9% Sigma-Aldrich, USA) was used to analyze changes in ionic strength.

GMA (97%, Aldrich, MO, USA) was used as a precursor for PGMA; MBAAm (99%, Sigma-Aldrich, USA) and benzoyl peroxide (BzO_2_, 25% H_2_O, Merck) were used as crosslinkers and initiators, respectively. In addition, poly(vinyl alcohol) (PVA; Mw: 30 kDa, 100% hydrolyzed Sigma-Aldrich, USA) was used as a stabilizing agent, and isopropyl alcohol (99%, Aldrich, USA) was used as a cosolvent. Double-distilled water was used as the solvent in all cases. In addition, absolute methanol (Aldrich, USA) was used as a washing solvent in the purification of the polymer. PEI (PEI; branched, M_n_ ≈ 10 kDa; Sigma-Aldrich) was used to introduce amino groups by grafting after obtaining PGMA microspheres.

### 2.2. Synthesis of MgONPs and CaCO_3_NPs

MgONPs were prepared from the calcination of a certain amount of Mg(OH)_2_ (Merck) at 500 °C for 2 h, followed by its subsequent disintegration by mechanical grinding [29].

(R1) $$Mg{\left(OH\right)}_{2}\left(s\right)\to MgO\left(s\right)+H_{2}O↑\left(g\right)$$

CaCO_3_NPs were obtained by a double displacement reaction (or salt metathesis reaction) followed by controlled precipitation of particles. For this, a mixture of two solutions was prepared: the first consisted of a 10% *w*/*v* mixture of NaCO_3_ (0.1 mol/L) and NaOH (0.2 mol/L), while the second was a 0.1 mol/L CaCl_2_ solution. CaCl_2_ solution was kept under constant stirring at 1400 rpm and 80 °C, while the first solution was added dropwise to promote controlled precipitation of CaCO_3_. Corresponding reaction is given by:

(R2) $${Na}_{2}{CO}_{3}\left(ac\right)+Ca{Cl}_{2}\left(ac\right)\to Ca{CO}_{3}\left(s\right)+2NaCl\left(ac\right)$$

Once the reaction was completed, the resulting mixture was filtered, washed with plenty of water, dried at room temperature, and mechanically crushed using a mortar [30].

### 2.3. Synthesis of the Crosslinked PGMA

#### 2.3.1. Synthesis of PGMA Particles

PGMA synthesis was evaluated using two methods: bulk polymerization and suspension polymerization, following the methodology described by Donia et al. (2006) [31] and Costa et al. (2020) [32]. For suspension polymerization, an aqueous phase was prepared consisting of 9.1 mL of a PVA solution (1% *w*/*w*), which was mixed with GMA (1.2 mL), MBAAm (0.0134 g), BzO_2_ (0.0210 g), and isopropyl alcohol (124 µL). The molar percentage of MBAAm with respect to the moles of GMA was 0.96%; whereas the molar percentage of BzO_2_ was 0.009%. The mixture was subjected to reaction in a reflux system at 80 °C with constant stirring at 350 rpm for 2 h. Once the reaction was complete, the polymer obtained was filtered and washed with hot deionized water (70 °C), followed by washing with methanol and finally dried in an oven at 50 °C. This procedure was repeated by increasing the amount of crosslinker (0.0066 g, 0.0134 g, 0.0199 g and 0.0268 g) to vary the crosslinking density and the material properties. On the other hand, bulk polymerization was performed using the same procedure and quantities previously described, but without the presence of the aqueous phase.

To evaluate the effect of different InAds on particle characteristics, MgONPs (N1: 0.0135 g, N2: 0.0453 g, and N3: 0.0906 g) and CaCO_3_NPs (N1: 0.0135 g, N2: 0.0453 g, and N3: 0.0906 g) were added to the aqueous phase in separate experiments during emulsion polymerization; where N1, N2, and N3 denote the levels of incorporation by mass. Likewise, the effect of ionic strength was evaluated by adding NaCl (N1: 0.0135 g, N2: 0.0453 g, and N3: 0.0906 g, where N1, N2 and N3 denote the level used in each case).

#### 2.3.2. PEI Chain Grafts on PGMA Spheres

In a 50 mL round-bottom flask, MgONP-doped PGMA microspheres (0.2006 g) were suspended in absolute ethanol (2.0 mL). Subsequently, PEI (1.04 g) was added to the suspension. Then, the mixture was refluxed at 70 °C with stirring for 10 h; finally, the mixture was filtered and washed with methanol and deionized water several times and dried in an oven at 50 °C for 10 h [12].

#### 2.3.3. Analysis of the Effect of InAds on Aqueous PVA Solutions

Aqueous PVA solutions (1% *w*/*v*) were prepared; from each, 9.1 mL was used per experiment. In independent systems, NaCl (soluble), MgO nanoparticles (MgONPs), and CaCO_3_ nanoparticles (CaCO_3_NPs) were added at four dosage levels: 0.0135, 0.0453, 0.0906, and 0.1353 g (1.48, 4.97, 9.95, and 14.87 mg mL^−1^). For MgONPs and CaCO_3_NPs, the solids were dispersed by stirring followed by brief sonication (ultrasonic bath, 3–5 min). After 10 min of homogenization, the dispersions (along with a PVA control without added salt) were analyzed by dynamic light scattering (DLS) using a Zetasizer Lab instrument (Malvern Panalytical) at room temperature, recording the hydrodynamic radius (Rh) and the polydispersity index (PDI). Each experiment was performed in triplicate.

#### 2.3.4. PGMA–(InAds)–PEI Resin

In a 50 mL round-bottom flask, 0.2006 g of PGMA–NaCl microspheres (N1, NaCl) were suspended in 2.0 mL of ethanol (absolute grade, Merck), and 1.04 g of PEI were added. The mixture was refluxed at 70 °C under stirring for 10 h to induce the reaction. The resulting material was then filtered, washed several times with ethanol and deionized water, and dried in an oven at 50 °C for 10 h. This procedure was repeated with MgONPs and CaCO_3_NPs.

### 2.4. Characterization Analyses

#### 2.4.1. Infrared and UV–Vis Spectroscopic Characterization

The materials were characterized by Fourier transform infrared spectroscopy using the attenuated total reflectance mode (FTIR–ATR, IRAffinity-1S, Shimadzu Co., Kyoto, Japan) in the spectral range of 4000–600 cm^−1^. UV–Vis spectroscopy was performed in the 200–800 nm region using a V-750 spectrophotometer (Jasco, Kyoto, Japan) equipped with an ISV-922 integrating sphere (Jasco, Kyoto, Japan).

#### 2.4.2. Thermal Characterization

Approximately 4.0 mg of PGMA samples were weighed for differential scanning calorimetry (DSC) analysis using a DSC25 (TA Instruments). Measurements were carried out from an initial temperature of 25 °C to 250 °C at a heating rate of 10 °C min^−1^. PGMA samples before and after PEI incorporation were further characterized by thermogravimetric analysis (TGA). The analyses were conducted from 20.0 °C to 600.0 °C at a heating rate of 10.0 °C min^−1^ under a nitrogen (N_2_) atmosphere.

#### 2.4.3. Morphological Characterization

The resins were examined by scanning electron microscopy (SEM) (Thermo Fisher Scientific Inc., Waltham, MA, USA) and energy-dispersive X-ray spectroscopy (EDS) (Thermo Fisher Scientific Inc., Waltham, MA, USA) using a Phenom Pro-X instrument under standard holder conditions at an accelerating voltage of 15 kV. Additionally, the resins were observed using an optical microscope (Leica Microsystems, Deerfield, IL, USA) with a 4× objective by dispersing the material onto a sample holder.

#### 2.4.4. Determination of Water Absorption Capacity (WAC)

The WAC of the obtained materials was determined gravimetrically following the method reported by Zhang et al. (2020) [33]. First, the water retention percentage of the funnel–membrane system (polypropylene membrane, Delvstlab, Haining, China, 0.45 µm) was determined after water suction through the membrane placed in a Büchner funnel under vacuum. For each resin, 0.12 g of sample was immersed in 25 mL of deionized water and allowed to interact for 5 min and 1 h. Subsequently, the water–resin mixture was transferred onto the preconditioned membrane (previously wetted with 10 mL of water) placed in the funnel under vacuum. Several washing steps were performed to prevent material loss. After 30 s of suction and confirmation of the absence of residual water, the vacuum pump was turned off. Any remaining water traces were removed by capillary action using lint-free absorbent paper. The mass of the wetted membrane–funnel assembly ($M_{EM}$), the mass of the dry resin ($m_{r}$), and the mass of the wetted membrane–funnel–wet resin assembly ($M_{EMr}$) were used to calculate the percentage of water retained by the resin as follows:

(1) $$CAA\left(\%\right)=\frac{M_{EMr}-M_{EM}-m_{r}}{m_{r}}\times 100.$$

#### 2.4.5. Determination of Epoxy Group Content

The content of residual free epoxy groups in the PGMA resins was determined by calculating the number of moles of epoxy groups per gram of sample (EV) using a back-titration method. Briefly, 0.030 g of each material was reacted with a solution of HCl (37%, Merck) in acetone (Merck) at a 1:40 v/v ratio (HCl:acetone). A standardized 0.10 mol L^−1^ NaOH solution was used as the titrant. The EV value was calculated according to He et al. (2014) [34] using the following equation:

(2) $$EV=\frac{\left(V_{0}-V\right)\times N}{W},$$

where $V_{0}$ is the volume of NaOH consumed in the blank (in L), V is the volume of NaOH consumed in the sample (in L), N is the molarity of the standardized NaOH solution (mol L^−1^), and W is the mass of the sample (g). Additionally, for the PEI-g-(MgO/PGMA) resin (i.e., resin loaded with MgO and grafted with PEI), the total functional group loading (${CA}_{Total}$) related to epoxy and amine groups was determined using the same procedure [34].

#### 2.4.6. Adsorption Experiments of ACT, AR-27, and ${NO}_{2}^{-}$

The adsorption capacity of MgO/PGMA and PEI-g-(MgO/PGMA) resins toward ACT and AR-27 was evaluated using a batch adsorption method. The effects of contaminant concentration, adsorbent dosage, contact time, pH, and ionic strength were investigated. For concentration studies, 10.0 mL solutions at 1.0, 2.0, and 3.5 ppm were prepared. Subsequently, 0.05 g of MgO/PGMA was added; in a second set of experiments, 0.05 g of PEI-g-(MgO/PGMA) was used. The mixtures were stirred for 1 h.

The effect of contact time was evaluated using 10.0 mL of 1.0 ppm adsorbate solutions with 0.05 g of adsorbent at interaction times of 30 min, 1 h, and 2 h under constant stirring. The effect of adsorbent dosage was assessed using 0.05 g, 0.10 g, and 0.20 g of material in 10.0 mL of 1.0 ppm contaminated solution with 1 h of agitation. The effect of pH was examined using 3.5 ppm solutions adjusted to pH 4.0 and pH 9.0; in these experiments, 0.10 g of adsorbent was used with 1 h of stirring. Finally, the influence of ionic strength was studied at three levels: 6.35 × 10^−6^ m (F1), 0.01 m (F2), and 0.70 m (F3), prepared by adding NaCl to 3.5 ppm adsorbate solutions. In these essays, 0.10 g of adsorbent was again used with 1 h of interaction under stirring. In all cases, the residual adsorbate concentration was determined by UV–vis spectroscopy. Calibration curves for Act and *AR-27* were previously prepared and measured at 242 nm and 521 nm, respectively.

The maximum retention capacity (*MRC*) and removal percentage (R) were calculated according to the following equations:

(3) $$MRC=\frac{\left({C_{i}V}_{i}-{C_{f}V}_{f}\right)}{M},$$

(4) $$R=\left(1-\frac{C_{f}V_{f}}{C_{i}V_{i}}\right)\times 100,$$

where $C_{i}$ and $C_{f}$ are the initial and final contaminant concentrations, respectively; M is the mass of adsorbent (g); and $V_{i}$ and $V_{f}$ are the initial and final solution volumes, respectively.

For ${NO}_{2}^{-}$, an exploratory analysis was performed using 0.05 g of resin and 10.0 mL of ${NO}_{2}^{-}$ (1.0 ppm), and the effect of pH was evaluated considering a resin–analyte interaction time of 0.5 h in aqueous phase. The corresponding R and MRC values were calculated. Determination of ${NO}_{2}^{-}$ in aqueous solutions was performed using the diazotization method (Standard Methods, 4500-NO_2_^−^ B, Colorimetric Method), based on the formation of a diazo complex with the analyte. A 1000 ppm NaNO_2_ (Supelco) stock solution was prepared, from which a working solution (10.0 ppm) was obtained. After, a calibration curve was constructed in the range of 0.01–0.125 ppm. In addition, the azo color reagent was prepared by dissolving 0.5032 g of sulfanilamide (≥98.0%, Merck), 0.0053 g of N-(1-naphthyl)ethylenediamine·2HCl (≥98.0%, Merck), and orthophosphoric acid (≥85.0%, Merck) in 50.0 mL of deionized water. Finally, for color development, 5.0 mL of ${NO}_{2}^{-}$ solution (standard or sample) was mixed with 200.0 µL of the color reagent and allowed to stand for 40 min in amber containers prior to spectrophotometric measurement at λ = 542 nm.

#### 2.4.7. Desorption of AR-27 and ${NO}_{2}^{-}$

For *AR-27* and ${NO}_{2}^{-}$ desorption experiments, 0.05 g of PGMA-PEI resin was treated with 10.0 mL of deionized water adjusted to pH 11.0. Four consecutive extraction cycles were performed by adding fresh desorption solution until the complete disappearance of the spectroscopic signal of the analytes in the aqueous phase was observed.

## 3. Results and Discussion

### 3.1. Synthesis of MgONPs and CaCO_3_NPs

**Figure 3:**
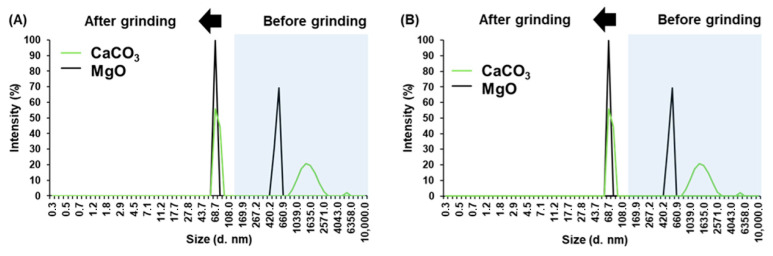
DLS analysis of MgO and CaCO_3_ before and after grinding ((**A**,**B**) are replicates).

The results of DLS analysis for MgO and CaCO_3_ particles are shown in Figure 3. In both cases, the milling process led to a significant reduction in hydrodynamic diameter. For CaCO_3_NPs, particle sizes decreased from an initial range of 1209.0–1901.0 nm to an average diameter of 68.69 nm. The observed size distribution was uniform and monodisperse, consistent with controlled precipitation followed by mechanical treatment. Similarly, MgONPs exhibited a reduction from an initial diameter range of 488.7–568.3 nm to final particle sizes between 68.69 and 79.88 nm. These results confirm the effectiveness of the milling process in producing nanoscale materials with narrow size distributions.

### 3.2. Effect of Polymerization Type and MBA Content on PGMA Synthesis

During PGMA synthesis, bulk polymerization produced glassy and irregular materials, consistent with the Trommsdorff autoacceleration effect, a phenomenon inherent to this synthesis method. This effect is characterized by a fast increase in viscosity and reduced radical termination rates, leading to the formation of dense polymeric bodies [35]. In contrast, aqueous suspension polymerization in the presence of PVA operates as a system of microreactors (organic droplets) stabilized at the interface by PVA. PVA prevents droplet coalescence and preserves sphericity, resulting in the formation of spherical beads.

**Figure 4:**
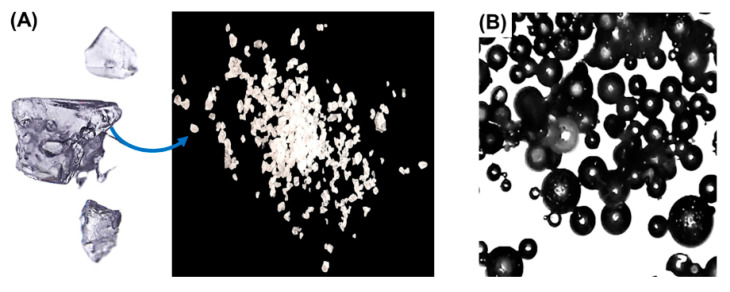
Effect on the morphology of PGMA resin using two synthesis methods: (**A**) bulk polymerization and (**B**) emulsion polymerization.

Suspension polymerization revealed a clear dependence of morphology on the concentration of the crosslinker MBA. As the MBA content increased from 0.0066 g to 0.0268 g, particle agglomeration increased and sphericity decreased, leading to heterogeneous and irregular morphologies (Figure 4). This morphological dependence on crosslinker concentration can be attributed to an increased probability of interparticle crosslinking during polymer growth. MBA exhibits moderate solubility in water, which increases with temperature. Because MBA is not completely confined to the GMA organic phase, the aqueous phase becomes enriched with crosslinker; consequently, the probability that growing polymer chains exceed the defined MBA/H_2_O interfacial boundary increases. Once this interfacial limit is surpassed, polymer chains propagate into the aqueous phase and interact with MBA, altering particle morphology. As a result, the system evolves from spherical particles defined by dispersed MBA droplets in the aqueous phase to irregular structures formed by polymer network growth followed by MBA particle agglomeration.

**Figure 5:**
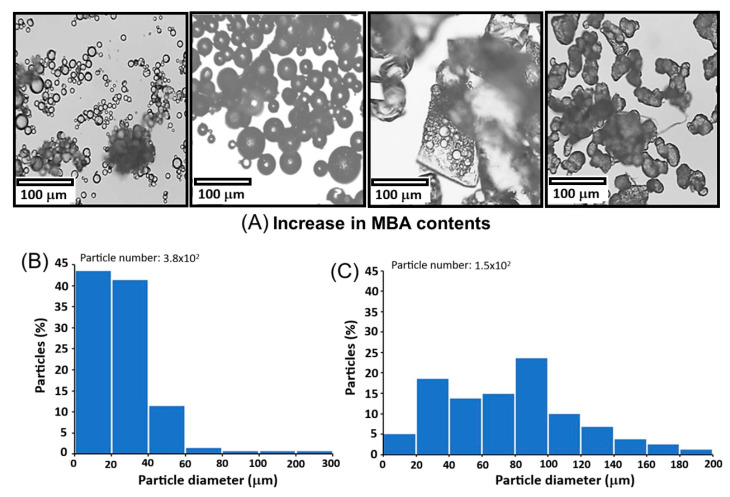
Effect of MBA concentration on PMGA morphology. Micrographs as a function of MBA content (**A**), histogram of PMGA particle diameters with 0.0066 g (**B**) and with 0.0134 g (**C**).

At low crosslinker concentrations, the polymer network is more flexible and less dense, limiting excessive particle growth and promoting interaction with the continuous phase of the suspension. This favors the preservation of spherical morphology without collapse [35]. Indeed, at the lowest MBA content (0.0066 g), 80% of the particles were smaller than 40 µm, whereas increasing the MBA amount to 0.0134 g resulted in larger particle sizes (85% showed a size between 20 and 120 µm). Conversely, higher crosslinker concentrations produce a more rigid and densely crosslinked polymer network, restricting stabilizer mobility. Under these conditions, gelation occurs more rapidly, prior to full spherical stabilization, thereby promoting irregular morphology and agglomeration (see Figure 5).

### 3.3. PGMA Resins Obtained by Suspension Polymerization with Addition of NaCl, MgONPs and CaCO_3_NPs

#### 3.3.1. Effect of the Aqueous-Phase Saline Composition on Suspension Polymerization

To elucidate the role of the addition of InAds on the colloidal stability of the system, model 1% *w*/*v* PVA solutions were analyzed by DLS at 25 °C in the absence and presence of NaCl, MgONPs, and CaCO_3_NPs. Although these conditions do not reproduce the polymerization temperature (80 °C), they allow evaluation of the supramolecular organization of PVA chains in the presence of each additive prior to reflux with the remaining polymerization reagents.

**Figure 6:**
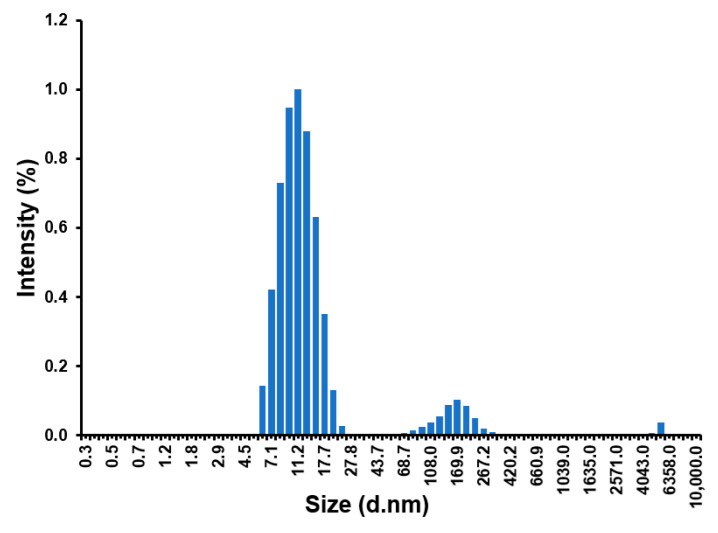
DLS of PVA in aqueous solution (1% *w*/*v*).

As shown in Figure 6, in the absence of additives, the PVA solution exhibits an essentially bimodal size distribution, with a dominant population centered at approximately 11 nm. This suggests that individual polymer chains adopt a solvated coil conformation. A secondary, lower-intensity population was observed in the 100–300 nm range, consistent with the formation of metastable supramolecular aggregates induced by strong intermolecular hydrogen bonding between the abundant hydroxyl groups of fully hydrolyzed PVA [36].

The distinct DLS responses obtained for PVA solutions in the presence of NaCl, MgONPs, and CaCO_3_NPs can be qualitatively rationalized in terms of specific ion effects (i.e., Hofmeister series). Thus, in macromolecular systems, the Hofmeister series describes how different anions and cations modulate water structure, polymer and protein solvation, and salting-in/salting-out phenomena beyond a simple increase in ionic strength [37,38].

**Figure 7:**
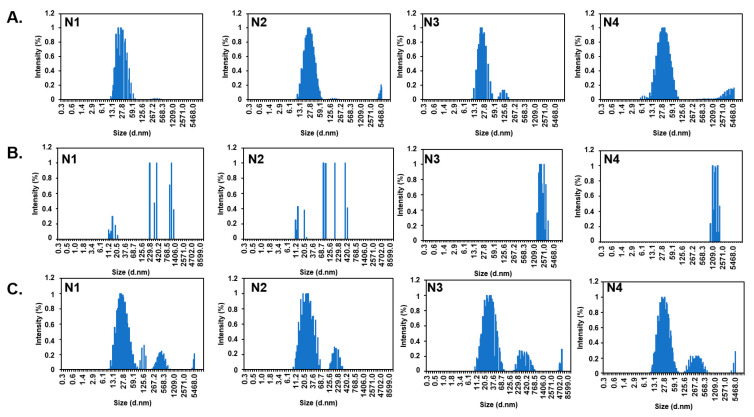
Effect of NaCl (**A**), MgONPs (**B**) and CaCO_3_NPs (**C**) on the size distribution of PVA (1% *w*/*v*) in aqueous solution (DLS, 25 °C). N1: 0.0135, N2: 0.0453, N3: 0.0906 and N4: 0.1353 g.

For neutral hydrophilic polymers such as fully hydrolyzed PVA, intermediate ions in the Hofmeister series (e.g., Na^+^/Cl^−^) typically induce relatively modest changes in chain hydration and conformation. In contrast, more strongly kosmotropic anions (i.e., small, highly charge-dense species such as SO_4_^2−^ and CO_3_^2−^) as well as certain divalent cations, can promote partial desolvation and aggregation processes. Consistent with this framework, PVA + NaCl solutions, where Na^+^ and Cl^−^ are fully dissolved in the aqueous phase, display a size distribution that remains largely unaltered, with hydrodynamic diameters in the ~10–30 nm range and only a very minor contribution at larger sizes (Figure 7A). This behavior agrees with the relatively intermediate character of Na^+^/Cl^−^ within the Hofmeister series [31], resulting in only modest perturbations of polymer hydration.

On the other hand, when CaCO_3_NPs and MgONPs were introduced, the system became more complex. In the case of CaCO_3_NPs, a fraction of the solid remains undissolved in the aqueous phase, whereas MgO reacts with water to generate Mg^2+^ and OH^−^ ions. For PVA in the presence of CaCO_3_NPs, the size distributions suggest coexistence of individual polymer coils and larger clusters of PVA chains, likely promoted by salting-out effects associated with the presence of released CO_3_^2−^ ions. However, the formation of hybrid PVA–CaCO_3_ aggregates is also plausible, in which PVA adsorbs onto the nanoparticle surface. This interpretation is consistent with the observed increase in hydrodynamic radius (see Figure 3, Figure 6 and Figure 7B) and previous reports describing polymer adsorption onto carbonate surfaces [36].

In the case of PVA in the presence of MgONPs, the DLS data exhibit a markedly broader and apparently “chaotic” distribution, characterized by a significant shift toward sub- and micrometric aggregates and a pronounced decrease in the fraction of PVA with small Rh. Rather than attributing this behavior solely to classical Hofmeister effects, it is more reasonable to consider the combined influence of: (i) the high basicity of the MgO/Mg(OH)_2_ surface, (ii) possible intramolecular coordination of Mg^2+^ with PVA hydroxyl groups, (iii) interchain bridging phenomena mediated by Mg^2+^ coordination, and (iv) adsorption of PVA molecules onto MgONP surfaces. The latter mechanism has been previously reported in PVA/MgONP-based films, where nanoparticles are stabilized through the formation of a surrounding PVA coating layer [36].

#### 3.3.2. Relationship Between Aqueous-Phase Composition and PGMA Morphology

**Figure 8:**
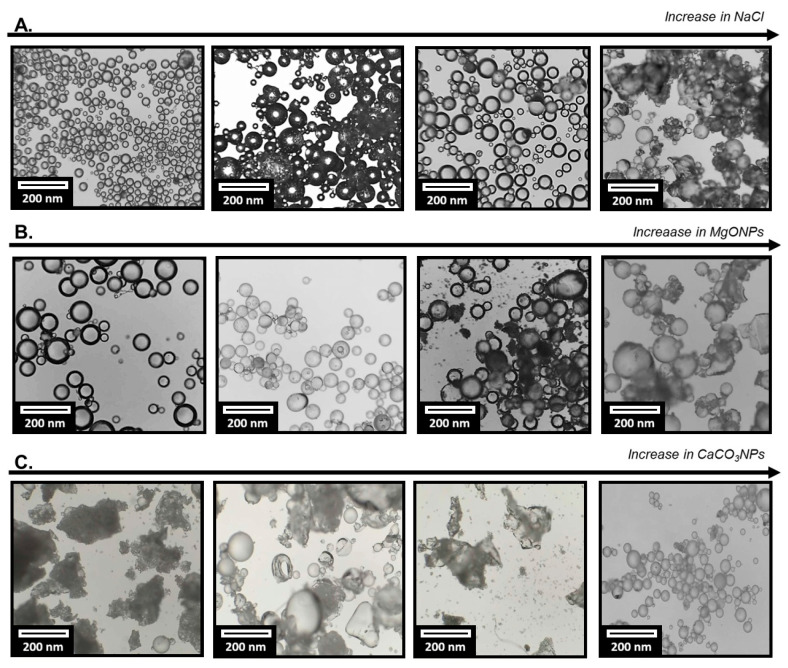
PGMA microspheres prepared with the addition of NaCl (**A**), MgONPs (**B**) and CaCO_3_NPs (**C**) in the aqueous phase. From left to right, increasing amount of solute (N1: 0.0135, N2: 0.0453, N3: 0.0906 and N4: 0.1353 g).

In systems formulated with NaCl (Figure 8A), a morphology predominantly composed of microspheres was observed in all cases. As salt concentration increased, a rise in polydispersity and a slight tendency toward bead coalescence or interparticle contact were noted, particularly in formulation N4. Nevertheless, the overall structure remained that of discrete particles. This behavior is consistent with the previously discussed DLS results (Figure 7A), where NaCl addition did not significantly alter the conformation of PVA chains. Under the evaluated conditions, most PVA molecules remain available as molecular stabilizers at the water/monomer interface, thereby preserving the classical steric stabilization mechanism characteristic of suspension polymerization [35]. In contrast, for the PVA–MgONP system, and considering the interactions described in Section 3.3.1, the presence of nanoparticles likely modifies the microstructure and mechanical properties of the matrix [37,38]. Partial sequestration of PVA in the vicinity of MgONPs reduces the fraction of polymer available to uniformly coat the monomer/water interface. This interpretation is consistent with the appearance of aggregated and irregular domains in the corresponding resins.

For the CaCO_3_NP system, low and intermediate inorganic loadings led predominantly to flocculated structures and irregular fragments, with only a small fraction of well-defined microspheres. Interestingly, a relatively homogeneous population of spherical particles was recovered only at the highest CaCO_3_ content (N4). At low to moderate inorganic concentrations, these observations may correlate with the bimodal DLS behavior described in Section 3.3.1 (Figure 7C), where free PVA coils coexist with PVA–CaCO_3_ clusters of approximately 10^2^ nm. The presence of such hybrid domains likely affects the emulsifying efficiency of PVA, since a significant fraction of the stabilizer becomes incorporated into polymer–nanoparticle aggregates. This redistribution alters interfacial organization and reduces monomer droplet stability, ultimately leading to fragile and flocculated structures.

Conversely, at the highest CaCO_3_ loading (N4), a saturation regime may be reached in which emulsion stabilization proceeds via solid-particle (Pickering-type) stabilization [39,40]. Under these conditions, a dense particulate layer forms around the monomer droplets, acting as a mechanical armor. This effect is further reinforced by the increased viscosity of the aqueous phase, which minimizes disruptive droplet collisions during agitation.

Overall, based on the combined information from Figure 7 and Figure 8, it can be concluded that the way NaCl, MgONPs, and CaCO_3_NPs reorganize PVA chains in solution at 25 °C provides insight into how these additives influence PGMA resin morphology at 80 °C. When PVA chains remain predominantly as free solvated coils, well-defined microspheres are obtained. In contrast, when a fraction of the polymer is incorporated into hybrid domains with nanoparticles (MgONPs or CaCO_3_NPs), stabilization of the organic droplets becomes less efficient, leading to more aggregated or poorly defined morphologies. This effect is particularly pronounced for CaCO_3_NPs at low and intermediate concentrations.

An increase in ionic strength leads to a decrease in the hydrodynamic radius (R_h_), as it promotes polymer–polymer interactions by reducing water structure (salting-out effect) and diminishing the hydration of PVA chains. In the case of monovalent ions, no specific interactions with PVA are expected; therefore, the decrease in Rh is typically moderate. Furthermore, these ions do not significantly contribute to light scattering, and thus the DLS results can be primarily attributed to the polymer and the changes in its hydrodynamic radius with increasing ionic strength [38]. In contrast, divalent ions produce a more pronounced effect. Beyond the increase in ionic strength, they induce stronger dehydration of PVA chains and can form intermolecular complexes, leading to the development of polymer-rich domains (i.e., aggregates). In DLS measurements, this behavior is reflected as an apparent increase in Rh; that is, despite enhanced intramolecular interactions, intermolecular associations between polymer chains are favored, ultimately promoting aggregate formation and an increase in the measured hydrodynamic radius [39]. These effects reflect a shift in the balance between polymer–water and polymer–polymer interactions, ultimately governing the conformational state of PVA in solution.

At room temperature, PVA–water interactions are sufficiently strong to disrupt the intramolecular hydrogen bonding interactions that occur within PVA chains; consequently, intermolecular interactions with water molecules are predominant [40]. The increase in polymerization temperature ($T_{pol}$) during the emulsion polymerization of GMA significantly influences both the conformation of PVA in the aqueous phase and the aggregation behavior of the resulting PGMA particles. Though $T_{pol}$ was relatively small with respect to room temperature (~55 °C), the effect is sufficient to produce significant changes in the system [41]. As temperature rises, intra- and intermolecular hydrogen bonding interactions between PVA chains and water molecules are progressively weakened, leading to a more flexible and expanded chain conformation; however, this also reduces the stability of the hydration layer surrounding the polymer, potentially decreasing its effectiveness as a steric stabilizer and promoting partial desorption or intermolecular aggregation at elevated temperatures [42].

On the other hand, with respect to polymerization, higher temperatures accelerate initiator decomposition and increase radical concentration, enhancing the polymerization rate but typically resulting in lower molecular weight PGMA chains. In our specific case, MBAAm produces the crosslinking required to avoid the dissolution of PGMA chains with low molecular weight. The increased thermal energy also promotes particle collisions and mobility within the emulsion droplets, and if PVA stabilization is compromised, this can lead to particle coalescence, broader size distributions, and reduced control over morphology [43,44]. Therefore, PVA concentration is identified as a necessary component of the polymerization system. In addition, to carry out this polymerization, the system composition should reflect a balance between enhanced reaction kinetics and diminished colloidal stability, where excessive temperature may ultimately favor aggregation phenomena over controlled particle formation.

#### 3.3.3. Morphological Characterization by SEM

**Figure 9:**
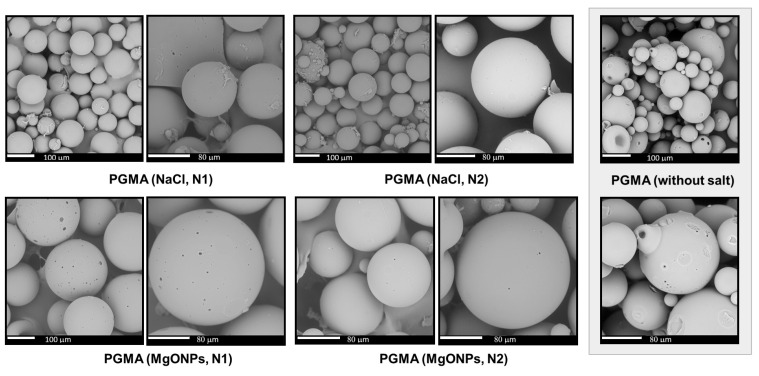
SEM analysis of PGMA microspheres obtained by adding two levels (N1 and N2) of NaCl and MgONPs in the PVA/H_2_O phase.

Figure 9 shows representative SEM micrographs of selected PGMA resins, specifically those prepared in PVA without salt addition and in the presence of NaCl and MgONPs at NaCl levels 1 and 2. The images confirm the spherical morphology of the PGMA particles and reveal the presence of surface cavities on the microspheres. However, in the absence of specific porosity characterization (e.g., N_2_ adsorption, Hg intrusion porosimetry, or tomography), it is not possible to unequivocally attribute these surface features to the presence of nanoparticles. Additional factors, such as gas bubble release during polymerization or differential polymer shrinkage during the drying stage, may also contribute to the formation of similar surface defects.

#### 3.3.4. Spectroscopic Characterization of PGMA

**Figure 10:**
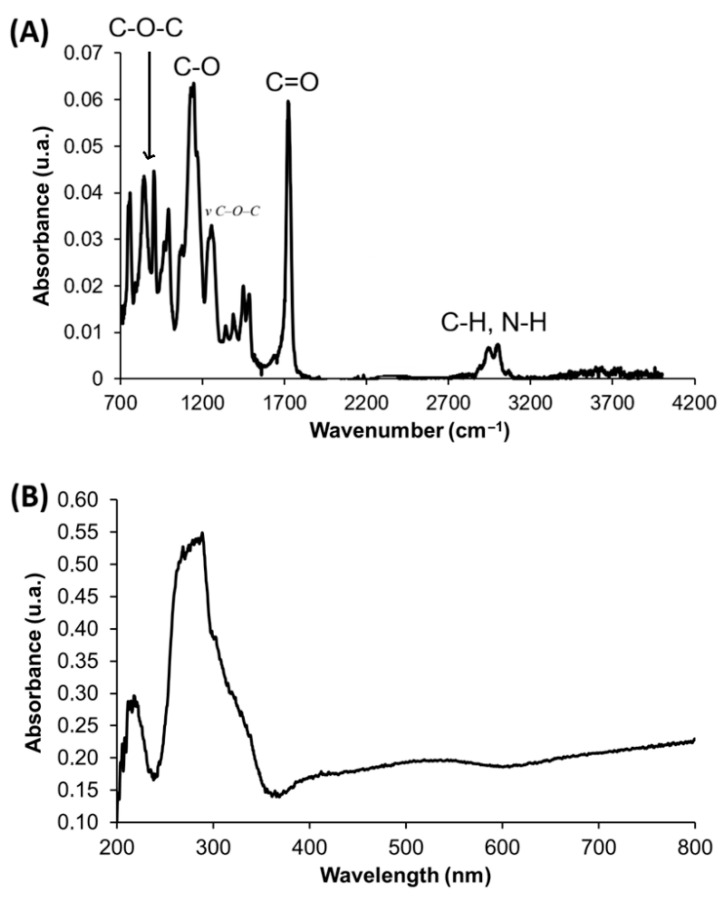
ATR-FTIR spectrum of PGMA resin (**A**) and UV-vis spectrum (**B**).

In general terms, all resins exhibited highly similar spectral profiles; consequently, Figure 10 presents representative FTIR and UV–vis spectra.

ATR–FTIR analysis shows a band at 3005 cm^−1^ associated with N–H stretching of the crosslinker. A band at approximately 2936 cm^−1^ corresponds to C–H stretching vibrations. The absorption at 1724 cm^−1^ is assigned to C=O stretching, while the signal at 1150 cm^−1^ corresponds to C–O stretching vibrations, confirming the presence of ester groups (–COO–). Additionally, bands at 1256 cm^−1^, 956 cm^−1^, and 843 cm^−1^ can be attributed, according to the literature [45], to C–O–C stretching vibrations of the epoxy ring, confirming the preservation of oxirane functionalities in the polymer structure.

The UV–Vis spectra display the characteristic electronic transitions reported for PGMA. In the 200–250 nm region, a band is observed corresponding to $\pi \;\to \;\pi^{*}$ transitions of the ester groups within the GMA backbone. A second intense band located between 250 and 350 nm is attributed to $n\;\to \;\pi^{*}$ transitions associated with epoxy and carbonyl groups.

#### 3.3.5. Thermal Characterization of PGMA by TGA and DSC

The thermograms of PGMA microspheres obtained in the absence and presence of NaCl, MgONPs, and CaCO_3_NPs during suspension polymerization exhibit nearly superimposable profiles. The thermal behavior is consistent with that reported in the literature for PGMA and GMA-based copolymers. An initial minor mass loss below approximately 120–150 °C is observed for all samples, attributable to the release of strongly retained water within the polymer network [44,45,46].

From approximately 280–300 °C onward, all samples display a single major degradation stage extending to ~420 °C. This stage is associated with depolymerization processes, random scission of the methacrylate backbone, and cleavage of ester and oxirane groups in PGMA. These observations are in agreement with the reported bulk decomposition ranges (~250–430 °C) for PGMA and porous poly(GMA-co-EGDMA) microspheres or GMA-based copolymers crosslinked with TRIM or 1,4-DMB [4,47].

**Figure 11:**
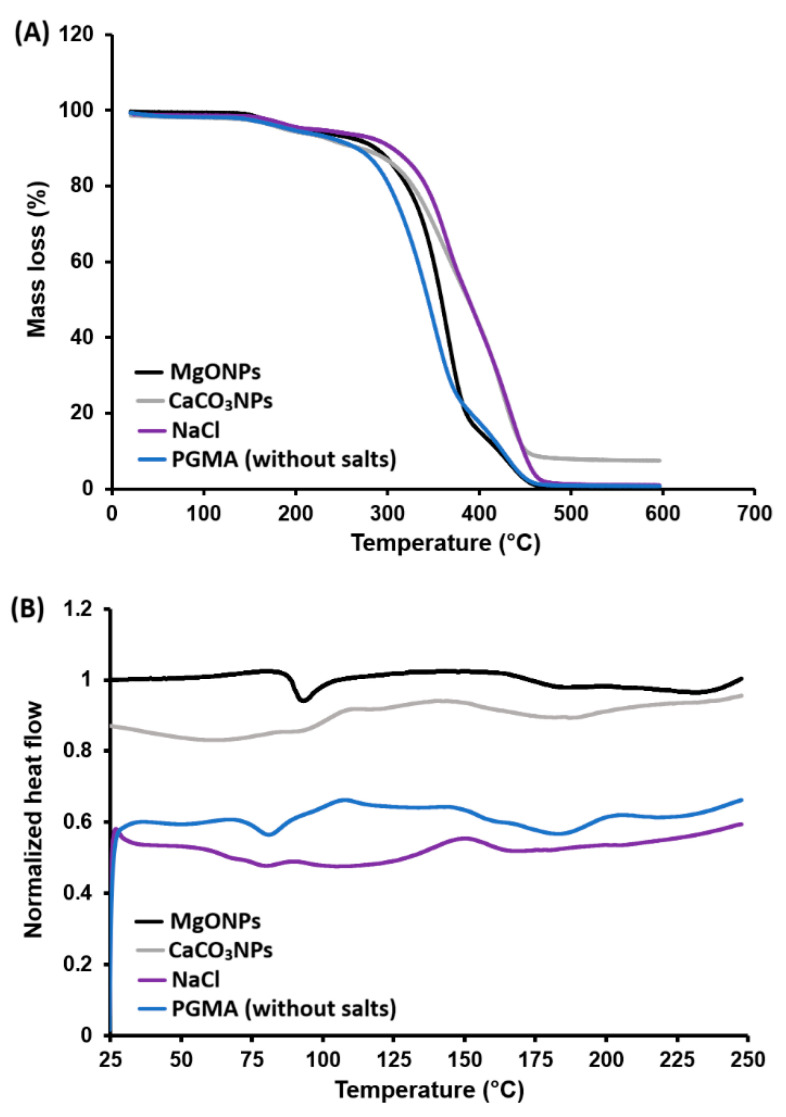
Thermograms of PGMA resins prepared with different salts in the aqueous phase (**A**), DSC analysis for PGMA (**B**).

The onset degradation temperatures and the slopes of the thermogravimetric curves are very similar across the four systems. The final residue is minimal and can be attributed to residual carbonaceous material and instrumental uncertainty. Assuming that any remaining inorganic fraction after washing is negligible, it can be concluded that the presence of salts or nanoparticles in the polymerization medium does not significantly affect the intrinsic thermal stability of PGMA. Previous studies have shown that PGMA obtained by solution polymerization exhibits relatively low glass transition temperatures. The TGA and DSC results for the materials developed in this work are presented in Figure 11.

The thermograms of the different PGMA resins (Figure 11) prepared in the presence of additives display a single broad thermal transition in the approximate range of 80–110 °C in all cases. In contrast, a Tg of 58 °C for PGMA synthesized by free-radical polymerization of GMA in benzene using AIBN, followed by purification via precipitation has been reported [48]. Similarly, Arslan et al. (2023) observed that Tg increased from undetectable values in low-molecular-weight samples to 75 °C and 78 °C in higher-molecular-weight and crosslinked samples, confirming that increasing molecular weight and chain entanglement shifts Tg toward higher temperatures [2].

Overall, the thermal profiles of the materials developed in this work remain comparable across formulations. Therefore, it can be inferred that the incorporation of NaCl or nanoparticles during suspension polymerization did not significantly alter the crosslinking density or the overall amorphous character of the PGMA matrix.

#### 3.3.6. WAC and EV of PGMA

**Figure 12:**
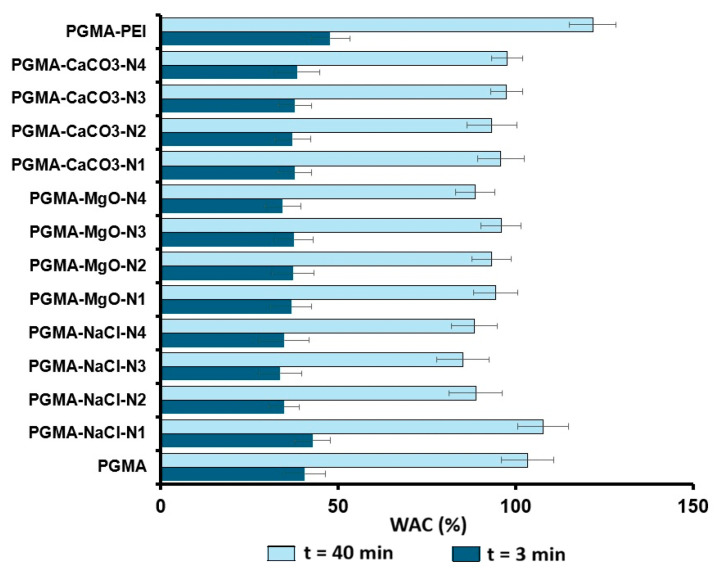
Values of WAC determined for the synthesized resins for 2 contact times (t) (t = 3 min and t = 40 min).

Figure 12 shows that the WAC of the modified PGMA microspheres remains within a relatively narrow range. After 40 min, WAC values lie approximately between 80 and 120%, whereas at 3 min they range from ~40 to 70%. Thus, all formulations retain comparable amounts of water. The differences observed among systems containing NaCl, MgO, and CaCO_3_ are not sufficiently pronounced to suggest drastic changes in overall water affinity.

**Figure 13:**
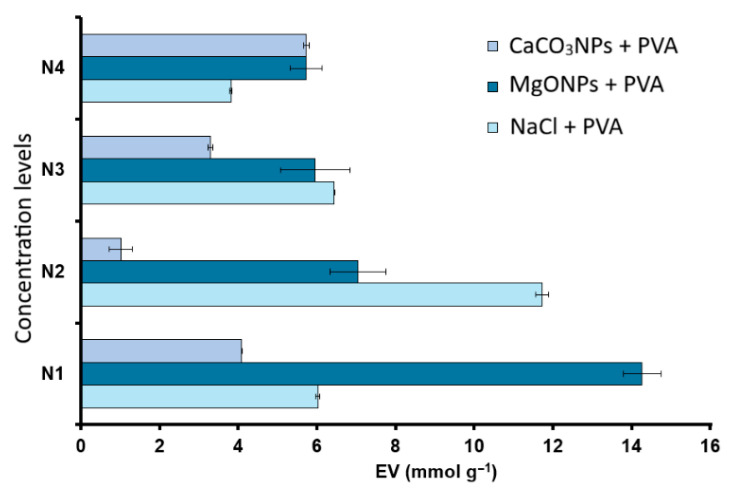
EV values determined for each type of PGMA resin synthesized by emulsion polymerization with PVA in the presence of salts (NaCl, MgONPs, CaCO_3_NPs) at four levels (N1: 0.0135, N2: 0.0453, N3: 0.0906 and N4: 0.1353 g).

In contrast, the EV exhibits a clear dependence on additive type and loading level (Figure 13). The resin synthesized without salt addition shows an EV of 4.83 ± 0.02 mmol g^−1^. Upon incorporation of additives into the aqueous phase, the epoxy group content of PGMA resins is influenced by both the nature of the additive and its concentration.

At the lowest level (N1), the MgONPs + PVA formulation exhibits the highest EV, with values approximately three times higher than the salt-free system, whereas NaCl + PVA and CaCO_3_NPs + PVA show more moderate increases. At N2, the NaCl + PVA system reaches the highest EV within the series, clearly exceeding the nanoparticle-containing formulations, while CaCO_3_NPs + PVA presents the lowest value. At N3, the differences between NaCl + PVA and MgONPs + PVA narrow, with similar EV values that remain higher than that of the salt-free resin, whereas CaCO_3_NPs + PVA remains in an intermediate range. Finally, at the highest loading level (N4), the trend reverses: CaCO_3_NPs + PVA exhibits the highest EV, followed by MgONPs + PVA, while NaCl + PVA shows the lowest value.

When these EV results are compared with morphological observations, it becomes apparent that formulations preserving discrete and relatively compact microspheres generally exhibit the highest epoxy group contents per gram of resin (e.g., MgONPs and NaCl at intermediate levels). In contrast, conditions under which microscopy reveals dense aggregates, partially collapsed particles, or fragmented structures, such as certain CaCO_3_NP loadings, correlate with lower EV values within the series. This correlation suggests that the same factors governing droplet stability during polymerization, namely PVA organization and viscosity, ionic strength, and the presence of basic solid surfaces, may also influence the preservation of epoxy rings. More stable dispersed phases may promote more homogeneous GMA polymerization with reduced epoxide ring opening. Conversely, conditions favoring droplet coalescence or particle collapse could be associated with a greater extent of side reactions involving epoxide ring opening. Additionally, the spherical configuration and the presence of dispersed surface cavities observed in some resins may contribute to the higher EV values measured in those systems.

Nevertheless, the relationship between morphology and EV is not strictly linear and should be interpreted carefully. Microscopy provides essentially structural information (particle size, shape, and surface texture), whereas EV represents a bulk chemical average of epoxy functionality throughout the resin volume. Therefore, although the observed EV variations are consistent with the reported morphological trends, additional factors, such as local pH, solvation microenvironments, and epoxy accessibility, are likely involved and cannot be inferred solely from morphological analysis.

### 3.4. Anchoring of PEI Groups onto PGMA

**Figure 14:**
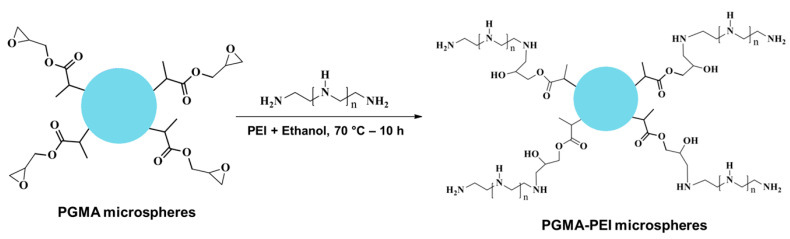
Reaction between PGMA microspheres and PEI.

Surface functionalization of PGMA microspheres was based on the high electrophilic susceptibility of the pendant oxirane ring toward nitrogen nucleophiles (see Figure 14). Modification with branched PEI proceeds via a nucleophilic ring-opening mechanism, in which primary and secondary amine groups of the polyelectrolyte attack the less sterically hindered carbon atom of the glycidyl group. This irreversible reaction generates stable covalent C–N bonds and leads to the formation of β-amino alcohol linkages at the interface and possibly within the microspheres. Owing to the hyperbranched architecture and high charge density of PEI, this strategy introduces active –NH_2_, –NH–, and –OH sites into the base resin, effectively converting it into a polycationic surface. Successful anchoring and the resulting evolution of the interfacial architecture are evidenced by the spectroscopic changes observed and by the enhanced retention capacity toward negatively charged species such as anionic dyes and ${NO}_{2}^{-}$ ions, behavior not exhibited by unmodified PGMA, as discussed below.

#### 3.4.1. Spectroscopic Characterization

**Figure 15:**
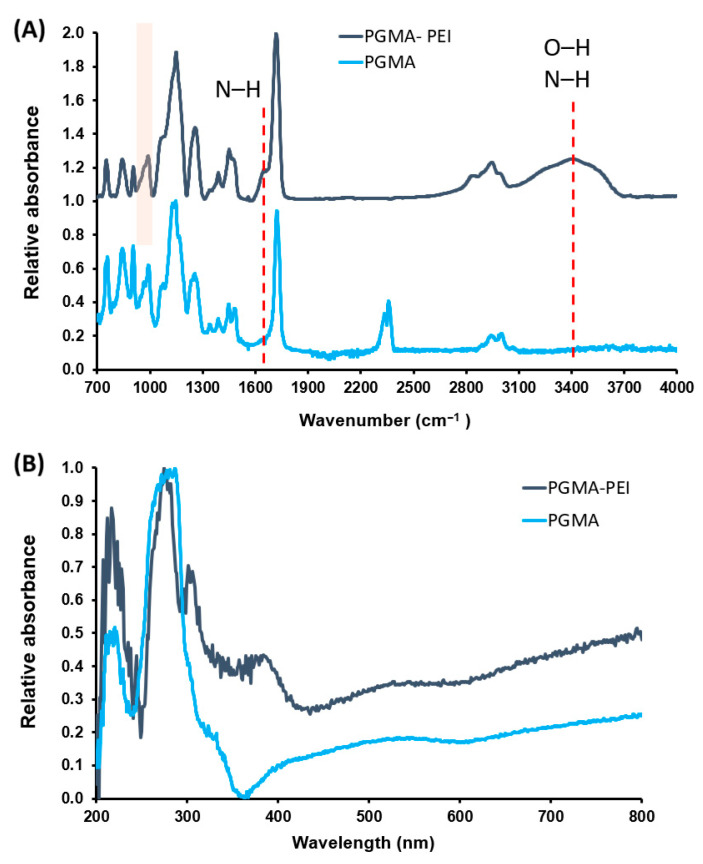
ATR-FTIR spectra: (**A**) UV-Vis spectra (**B**) for PGMA and PGMA-PEI (signals between 840–903 are related to partial conversion of epoxide groups into β-hydroxyalkyl amines).

The FTIR spectrum of the PEI-grafted PGMA spheres (PGMA–PEI) shows clear differences compared to unfunctionalized PGMA, consistent with epoxide ring opening and PEI chain anchoring onto the polymer matrix (Figure 15). The intense ester ν(C=O) band at approximately 1720–1730 cm^−1^ is preserved, indicating that the PGMA backbone remains essentially intact and that reaction with PEI occurs preferentially at epoxy groups rather than via extensive ester aminolysis. In contrast, the characteristic epoxy-ring bands in the 1250–1260 cm^−1^ region and, particularly, the signals at ~903 and 840 cm^−1^ decrease slightly in intensity relative to neat PGMA, consistent with partial conversion of epoxide groups into β-hydroxyalkyl amines. Additionally, PGMA–PEI exhibits a broader and more intense band in the 3200–3500 cm^−1^ region, attributable to overlapping ν(O–H) and ν(N–H) stretching vibrations generated after epoxide ring opening and due to the presence of primary and secondary amine groups from PEI. This modification is further reflected in the appearance or intensification of a band around 1600–1650 cm^−1^, associated with δ(N–H) bending vibrations, like those reported by Sun et al. (2013) [49]. The relative decrease in epoxy-related bands and the emergence of signals corresponding to amino and hydroxyl groups spectroscopically confirm the successful incorporation of PEI onto the surface of PGMA microspheres.

UV–Vis spectra of both resins exhibit the same general band pattern in the ultraviolet region, indicating that electronic transitions remain dominated by chromophores inherent to the PGMA matrix, as previously discussed. However, closer comparison reveals that PGMA–PEI shows slightly broader bands and subtle changes in the relative intensity of lower- and higher-wavelength maxima. Moreover, two additional signals appear in PGMA–PEI at 308 and 382 nm. These features may be associated with the introduction of PEI amino groups, whose lone electron pairs can interact with $n\;\to \;\pi^{*}$ transitions of carbonyl groups, thereby modifying the local electronic environment and giving rise to these additional absorptions.

#### 3.4.2. WAC, EV and SEM–EDS

**Figure 16:**
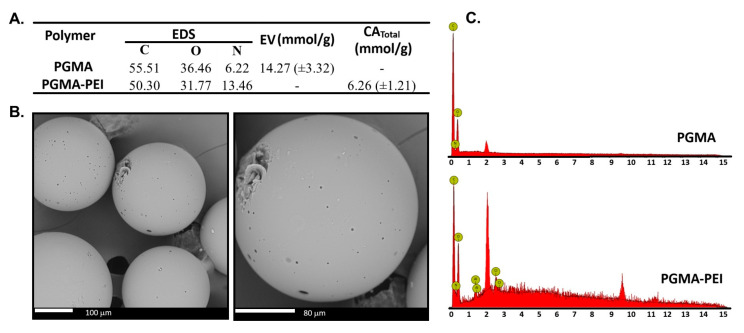
(**A**) Comparison of C, O and N composition by EDS, EV and Total Functional Content (CATotal) of the PGMA and PGMA-PEI, (**B**) SEM images of the PGMA-PEI resin and (**C**) EDS analysis.

As shown in Figure 16, PGMA–PEI exhibits a water retention capacity of 47.7% at 3 min and 121.8% at 40 min, representing an improvement in swelling capacity compared with the initial PGMA resins synthesized with MgONPs (N1). This result indicates that PEI incorporation promotes epoxide ring opening with the likely formation of β-hydroxyalkyl amines, introducing additional –OH and amine (–NH– or –NH_2_) groups per reactive site. These functionalities enhance hydrogen-bond formation with water, thereby increasing hydrophilicity. Regarding EV, the initial PGMA–MBA resin exhibited an EV of 14 mmol g^−1^ (according to the acid–base titration procedure employed). After PEI modification, the experimental value decreased to 6 mmol g^−1^.

For PGMA–MBA–PEI, this value no longer reflects exclusively residual epoxy groups but rather the combined contribution of all functionalities reacting with HCl under the assay conditions, particularly PEI amino groups in addition to any remaining epoxides. Therefore, the EV of the modified material should be interpreted semi-quantitatively.

Nevertheless, the marked decrease relative to the parent polymer indicates that a substantial fraction of glycidyl rings is no longer available, either due to reaction with PEI or to competing ring-opening processes (e.g., hydrolysis), confirming significant chemical modification of the PGMA–MBA matrix.

Complementary SEM–EDS analysis (Figure 16A,C) revealed a clear increase in nitrogen signal, rising from 6.22 wt% in unmodified PGMA–MBA resin to 13.46 wt% after PEI functionalization. It is important to emphasize that EDS is a semiquantitative technique with a limited analysis volume and no hydrogen detection capability; therefore, the reported mass fractions should be considered comparative indicators of nitrogen enrichment rather than absolute elemental compositions. However, the approximate 7.24% increase in nitrogen content is consistent with the incorporation of nitrogen-containing functional groups.

#### 3.4.3. Thermal Analysis: TGA

**Figure 17:**
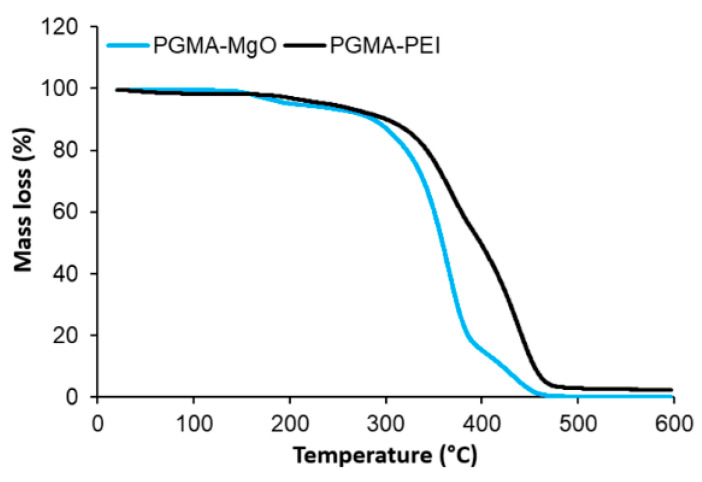
Comparison between TGA analyses for PGMA (with MgONPs, N1 = 0.0135 g in aqueous phase) and PGMA-PEI.

Figure 17 compares the thermograms obtained for unmodified PGMA and PGMA–PEI. Both resins remain thermally stable up to 250–270 °C, exhibiting only minimal mass losses attributable to residual moisture. Beyond this temperature, the main degradation stage of the polymers is observed. For neat PGMA (blue curve), mass loss begins at a slightly lower temperature and proceeds more abruptly, occurring predominantly between 290 and 410 °C. In contrast, the modified PGMA–PEI resin (black curve) shows a shift in the onset of degradation toward slightly higher temperatures (~320 °C), with a more gradual mass loss extending up to 470–500 °C. This displacement of the maximum decomposition region toward higher temperatures suggests that incorporation of PEI chains into the PGMA matrix confers a modest enhancement in thermal stability. This effect is likely associated with an increase in effective crosslink density and the formation of an extensive hydrogen-bonding network between PEI and the hydroxyalkyl groups generated upon epoxide ring opening. The final residue is low in both cases, although slightly higher for PGMA–PEI, consistent with the formation of a moderately more stable char promoted by nitrogen-containing functionalities.

### 3.5. Adsorption Behavior of AR-27 Dye and Act on PGMA–PEI

For AR-27 analysis, the calibration curve in the concentration range of 0.1–10 ppm yielded an R^2^ value of 0.985, with the linear regression equation: $y\;=\;0.0467x-8.49\times 10^{-5}$. For Act analysis within the same concentration range (0.1–10 ppm), the calibration curve presented an R^2^ of 0.989, corresponding to the regression equation: y = 0.0732x + 0.0764.

Before discussing the retention experiments, it is important to note that adsorption by unfunctionalized PGMA microspheres was negligible for AR-27, Act, and ${NO}_{2}^{-}$. Similarly, the PGMA–PEI resin did not retain ACT under any of the evaluated conditions. ACT is a weak acid with a carboxylic acid group (pKa = 9.5) [50]. Consequently, the carboxylic acid group on Act at pH < 9.5 is protonated, while the negatively charged carboxylate group predominates at pH > 9.5. In contrast, the PEI has a high positive charge density resulting from the protonation of amine groups (pKa ~7.2) [51]. This indicates that at the working pH, the electrostatic interaction mediated by the carboxylate (${-COO}^{-}$) and ammonium (${-NH}_{4}^{+}$) groups is not possible. Furthermore, other types of interactions, such as hydrophobic interactions resulting from the aromatic ring of Act, are not identified as significant due to the highly hydrophilic nature of the PEI. Furthermore, the formation of hydrogen bonds does not result in strong adsorption of acetaminophen onto PEI because of strong hydration (high competition of water for sites susceptible to hydrogen bond formation) and PEI’s greater affinity for water molecules. Additionally, the resin lacks extended aromatic domains or well-defined hydrophobic cavities capable of promoting π–π interactions or hydrophobic partitioning. Consequently, Act is more favorably stabilized in the aqueous phase through solvation rather than at the polymer surface. Accordingly, Act adsorption was negligible within experimental uncertainty.

#### Retention Experiments: AR-27

The adsorption kinetics of AR-27 (C_i_ = 1.3063 mg L^−1^) onto PGMA–PEI resin (0.050 g in 10 mL solution) at pH 7 were evaluated by fitting the experimental q_t_ data (0.139–0.253 mg g^−1^) to pseudo-first order, pseudo-second order, and intraparticle diffusion models. The time-dependent retention behavior is shown in Table 1. The experimental equilibrium adsorption capacity (q_e_) was determined to be 0.253 mg g^−1^, corresponding to the value reached after 3 h.

**Figure 18:**
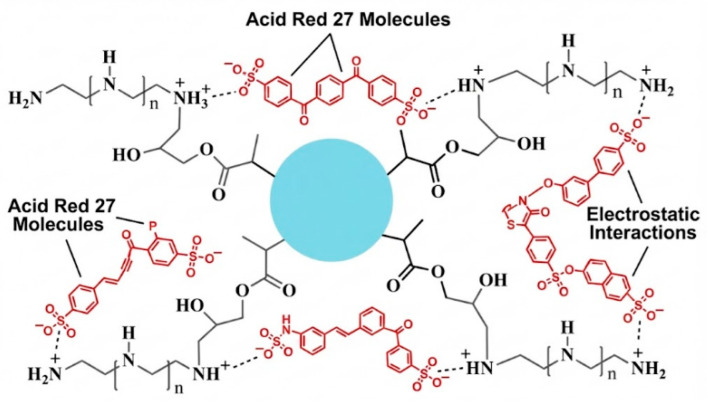
Interaction between the cationic groups of the PEI of PGMA-PEI and the negatively charged AR-27.

Table 2 summarizes the parameters obtained for each kinetic model evaluated. Largely, comparison of the kinetic models indicates that AR adsorption onto PGMA–PEI is more accurately described by the pseudo-second-order model, while also allowing for a contribution from intraparticle diffusion. These findings support a mechanism predominantly governed by specific electrostatic interactions between the protonated amine groups of PEI and the sulfonate groups of the dye (see Figure 18).

Increasing the initial AR-27 concentration from approximately 1.3 to 4.3 mg L^−1^ resulted in a decrease in removal efficiency from about 57% to values close to 44%, remaining essentially constant above 2.0 mg L^−1^ (Table 3). Under these conditions, the availability of cationic PEI sites becomes the limiting factor. As the dye concentration in solution increases, a progressively larger fraction of accessible ammonium groups in PGMA–PEI becomes occupied, eventually approaching saturation.

The pH of the medium exerted a significant influence on AR removal (see Table 4). At pH 4, the highest removal percentage (~77%) was achieved, which progressively decreased with increasing pH, reaching values on the order of 53% at pH 9. Since AR is a sulfonated azo dye that remains negatively charged throughout the studied pH range, the observed trend can be attributed to changes in the degree of protonation of the PEI chains anchored onto PGMA. Under acidic conditions, most amine groups (primary, secondary, and tertiary) are protonated, generating a high positive charge density and, consequently, strong electrostatic attraction toward the sulfonate groups of the dye. As the pH increases, a growing fraction of amine groups becomes deprotonated, reducing the density of available cationic sites and thus decreasing retention capacity.

Nevertheless, adsorption remains significant at neutral pH. At pH 9, a removal efficiency of approximately 50% suggests the presence of residual charged sites and/or additional contributions from hydrogen bonding and π–π interactions between the aromatic backbone of the dye and organic domains of the resin.

The ionic strength of the medium also had a pronounced effect on AR adsorption by PGMA–PEI (see Table 4). At low ionic strength (deionized water: 6.35 × 10^−6^ M), removal was in the range of 65–70%, decreasing to ~55% at 0.01 M and dropping almost completely (~5–10%) at 0.7 M. This marked dependence confirms that the fixation process is primarily governed by electrostatic interactions between protonated PEI amines and sulfonate groups of the dye. As ionic strength increases, electrolyte ions screen electrostatic attraction (through compression of the electrical double layer) and compete for charged sites, resulting in a substantial decline in adsorption efficiency at high salt concentrations. Additionally, PEI may adopt a more coiled conformation at higher ionic strength and elevated pH, as reported by De Britto et al. (2011) [52], due to charge neutralization and chain collapse. Such conformational changes could further hinder interaction with the negatively charged analyte.

AR-27 values using chitosan with different morphologies show relatively high adsorption values: chitosan with hydrogels (2732.2 mg g^−1^), aerogels (676.7 mg g^−1^), powders (534.8 mg g^−1^), and nanofibers (215.5 mg g^−1^) [53]. However, these materials are hydrogels, and therefore retention is not only mediated by surface adsorption but also by direct dye flow into the hydrogel as it reaches its maximum swelling capacity. On the other hand, adsorption on mineral surface is also relatively high; for example, illite (61 mg g^−1^), montmorillonite (30 mg g^−1^), and zeolite (4 mg g^−1^) [54,55,56,57]. This suggests that a high negative charge density is indicative of achieving high retention through adsorption. However, it is worth noting that key aspects such as pH and ionic strength are not analyzed in the cited studies, and, furthermore, the reported values correspond to results obtained from modeling using isotherms and not to continuous enrichment methodologies.

### 3.6. Adsorption of ${NO}_{2}^{-}$ on PGMA–PEI

**Figure 19:**
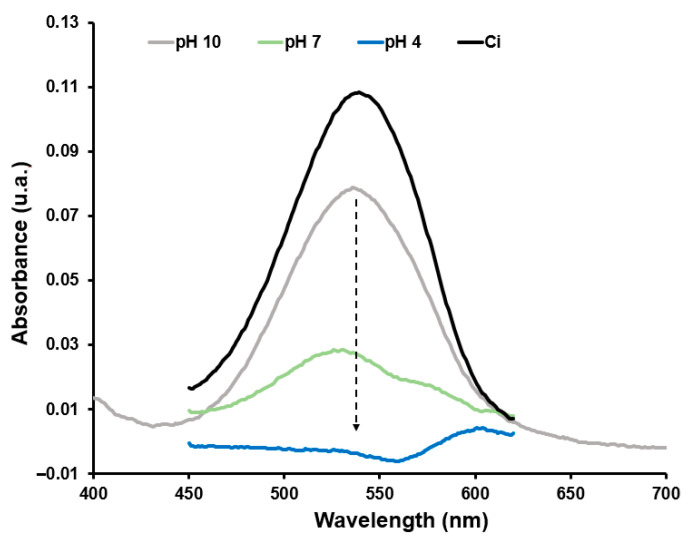
UV-Vis spectra of aqueous ${NO}_{2}^{-}$ solutions before ($C_{i}=1.0\;ppm$) and after contact with PGMA-PEI microspheres (0.05 g) for 0.5 h at pH 4.0, 7.0 and 10.0.

Determinations of ${NO}_{2}^{-}$ were performed using a calibration curve in the linear range of 0.01–0.125 ppm, yielding an R^2^ of 0.9975. All solutions were subjected to the diazotization reaction as described in the methodology. The retention behavior of the polymer toward this anion was observed for AR-27. The spectra shown in Figure 19 display a clear decrease in the characteristic absorption band of the colored ${NO}_{2}^{-}$ complex (540 nm) after contact with the PGMA–PEI microspheres, with the extent of reduction strongly dependent on pH. At pH 10, the final absorbance decreases only slightly relative to the initial solution ($C_{i}=1.0\;ppm$), indicating limited anion removal. At pH 7, the band intensity decreases much more markedly. Finally, at pH 4, the spectrum nearly collapses to the baseline, indicating almost complete removal of ${NO}_{2}^{-}$ from the aqueous phase after only 0.5 h of interaction with the resin. The overall trend is, therefore, an increase in ${NO}_{2}^{-}$ retention as pH decreases, consistent with a mechanism dominated by electrostatic interactions between the anion and protonated PEI amine groups, like that described for AR-27.

Quantitatively, Table 5 summarizes the calculated retention parameters. The maximum retention capacity (MRC) increases from 0.0063 mg g^−1^ at pH 10.0 to 0.0156 mg g^−1^ at pH 7.0 and reaches 0.0230 mg g^−1^ at pH 4.0, corresponding to removal efficiencies of 26.3, 70.8, and 100%, respectively. At pH 10.0, the behavior parallels that described for AR-27. As pH decreases toward neutral and slightly acidic conditions (pH 4.0), the degree of protonation of primary, secondary, and tertiary amines increases, generating a high density of ${-NH}_{3}^{+}$ sites capable of electrostatically binding nitrite anions, possibly reinforced by hydrogen bonding.

Overall, the behavior of PGMA–PEI toward ${NO}_{2}^{-}$ is consistent with that reported for other PEI-based anion adsorbents. The use of PEI-functionalized polyacrylonitrile fibers (PAN–PEI) for rapid ${NO}_{3}^{-}$ removal has been described, exhibiting pseudo-second-order kinetics and strong pH dependence attributed to amine protonation, closely resembling the trend observed here for ${NO}_{2}^{-}$ [58]. Similarly, it has been demonstrated that rice husk silica grafted with PEI shows high nitrate adsorption capacities governed by electrostatic interactions between protonated amines and oxyanions [59]. In addition, sodium alginate/branched PEI nanocomposite beads have been reported to follow the same retention mechanism, highlighting the sensitivity of the process to competing anions and to the degree of polymer protonation [60]. In this context, the high ${NO}_{2}^{-}$ removal efficiencies achieved with PGMA–PEI at pH 4.0–7.0 place the present material within the expected performance range of PEI-modified adsorbent systems.

### 3.7. Desorption Experiments

Since the materials were found to be unsuitable for acetaminophen removal, desorption experiments were conducted for AR-27 and ${NO}_{2}^{-}$.

#### 3.7.1. Desorption of AR-27

**Figure 20:**
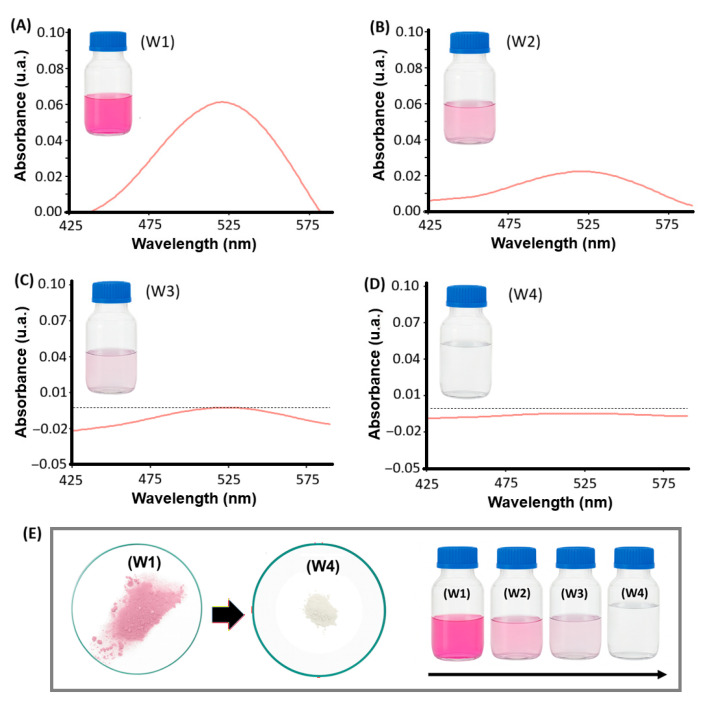
UV–vis spectra of the washing solutions for four desorption cycles using water (pH 11) (**A**–**D**), along with macroscopic visualization of changes in both the material and the washing solutions (W1, W2, W3, and W4 denote washing cycles 1, 2, 3, and 4, respectively). In (**E**) the change in color of the solid material from W1 to W4 is shown.

Figure 20A shows the UV–vis spectra of the washing solutions obtained over three desorption cycles. A progressive decrease in the characteristic absorption band of AR-27, centered at approximately 520 nm, can be observed. During the first washing step, the absorbance is high, and the solution exhibits an intense pink coloration, indicating that a significant fraction of the adsorbed dye is released at this stage.

This behavior is directly related to the regeneration conditions of the material. Since the washing is performed at pH 11, most of the amine groups of PEI are deprotonated under these conditions, and the resin no longer behaves as a cationic polyelectrolyte. As a result, electrostatic interactions are weakened, promoting dye desorption. In subsequent washing steps, the intensity of the absorption band decreases progressively until approaching the baseline, indicating that after a few cycles, the amount of AR-27 remaining on the material surface is minimal (see Figure 20B). Table 5 summarizes the results for two adsorption–desorption cycles, 1 and 2 (AD-1 and AD-2). It can be observed that as AR-27 desorption occurs, the material’s retention capacity decreases by approximately 24% (R changes from 47.6% to 36.2%). This suggests that regenerating the material with water at pH 11 leads to a loss of its adsorption capacity; however, it remains at around 75%, making its reuse possible. Nevertheless, further experiments should be conducted to establish the optimal regeneration conditions.

#### 3.7.2. Desorption of ${NO}_{2}^{-}$

Finally, each sample used in the previous experiments was regenerated by washing with four 10.0 mL portions of water adjusted to pH 10.0. The cumulative concentration measured in the washing solutions corresponded to 100% desorption of ${NO}_{2}^{-}$ from the resin (see Table 6).

## 4. Conclusions

Suspension polymerization of GMA in an aqueous medium stabilized with PVA enabled the preparation of MBA-crosslinked PGMA microspheres exhibiting predominantly spherical morphologies and good thermal stability. The incorporation of NaCl, MgONPs and CaCO_3_NPs into the aqueous phase revealed that each solute distinctly influences the organization of PVA. NaCl minimally perturbs the stabilizer structure, leading to uniform microspheres. In contrast, MgO promotes PVA aggregation, resulting in heterogeneous particles. CaCO_3_ induces flocculated structures at low loadings, whereas spherical morphology is recovered at higher concentrations, possibly due to a Pickering-type stabilization mechanism.

Functionalization of PGMA with PEI was successful, imparting increased hydrophilicity and positive surface charge. The resulting material exhibited high adsorption capacity toward anionic species (AR-27 and ${NO}_{2}^{-}$), following pseudo-second-order kinetics dominated by electrostatic interactions, along with effective regeneration under basic conditions.

It is concluded that PGMA-PEI microspheres are a promising alternative for the adsorption of anionic dyes such as AR-27 and inorganic anions such as nitrite. Specifically, it was observed that the nature of the interaction is primarily electrostatic; therefore, the reversibility of the process and the effect of the medium should be studied in future research.

## Figures and Tables

**Table 1:** Kinetic data of AR-27 adsorption on PGMA-PEI microspheres, 0.05 g; C_i_ = 1.31 mg L^−1^; V = 10 mL; pH 7.

Time (t)	C_f_	R	MRC	qt
(h)	(mg L^−1^)	(%)	(mg g^−1^)	(mg g^−1^)
0.5	0.61	53.25	0.138	0.14
1	0.56	57.11	0.148	0.15
2	0.05	96.36	0.250	0.25
3	0.04	96.92	0.251	0.25

**Table 2:** Kinetic parameters related to the evaluated models for the removal of AR-27.

Model	Parameters		R^2^
Pseudo-first order	k_1_ (h^−1^)	3.37	0.9031
	q_e_ (mg g^−1^)	1.16	
Pseudo-second order	q_e_	0.29	0.9434
	k_2_ (g mg^−1^ h^−1^)	4.27	
Intraparticle diffusion	k_id_ mg g^−1^ h^−1/2^)	0.1304	0.8790
	C (mg g^−1^)	0.04	

R^2^: Correlation coefficient.

**Table 3:** Variation in MRC and %R with the variation in the concentration of AR-27 in aqueous phase (Ci).

Ci	MRC	R
(mg L^−1^)	(mg g^−1^)	(%)
1.3	0.148	57.1
1.9	0.165	43.7
3.6	0.323	44.4
4.3	0.344	42.3

**Table 4:** Variation in MRC and R with the variation in ionic strength (IS) and pH in aqueous phase (Ci: 3.5 ppm of AR-27).

pH	IS	MRC	R
	(mmol L^−1^)	(mg g^−1^)	(%)
4	6.35 × 10^−6^	0.234	76.7
7	6.35 × 10^−6^	0.239	66.8
9	6.35 × 10^−6^	0.181	53.9
7	6.35 × 10^−6^	0.239	66.8
7	0.01	0.199	54.4
7	0.7	0.023	6.4

**Table 5:** AR-27 adsorption from PGMA-PEI microspheres for two-cycle adsorption–desorption.

pH	A_max_	[AR-27]	R
	(524 nm)	(mgL^−1^)	(%)
S0	0.1561	3.34	-
AD1	0.0818	1.75	47.6
AD2	0.0997	2.13	36.2

S0: Initial AR-27 dissolution in contact with PGMA-PEI microspheres. A_max_: Maximum absorbance.

**Table 6:** ${NO}_{2}^{-}$ retention by PGMA-PEI microspheres as a function of pH (MRC and R). Initial concentration of ${NO}_{2}^{-}=1.0\;ppm$.

pH	MRC	R
	(mg/g)	(%)
10	0.0063 ± 0.0005	26.25 ± 0.61
7	0.0156 ± 0.0002	70.83 ± 1.23
4	0.0230 ± 0.0001	100.00 ± 0.01

## Data Availability

The main data have been provided in this paper.

## Abbreviations

The following abbreviations are used in this manuscript:

ACT	Acetaminophen
AD-1 or -2	Adsorption–desorption cycles
A_max_	Maximum absorbance
AR-27	Acid red 27
ATR	Attenuated total reflectance
BzO_2_	Benzoyl peroxide
${CA}_{Total}$	Total functional group loading
CaCO_3_NPs	CaCO_3_ nanoparticles
$C_{i}$	Initial concentration
DSC	Differential scanning calorimetry
DLS	Dynamic light scattering
EDS	Energy-dispersive X-ray spectroscopy
EV	Epoxy value
FTIR	Fourier transform infrared spectroscopy
GMA	Glycidyl methacrylate
InAds	Inorganic additives
IS	Ionic strength
MBAAm	N,N′-methylenebisacrylamide
$M_{EM}$	Wetted membrane–funnel assembly (Equation (1))
$M_{EMr}$	Mass of the wetted membrane–funnel–wet resin assembly (Equation (1))
MgONPs	MgO nanoparticles
$M_{n}$	Average molecular weight in number
$m_{r}$	Mass of the dry resin (Equation (1))
MRC	Maximum retention capacity
M_w_	Average molecular weight in mass
PDI	Polydispersity index
PEI	Polyethyleneimine
PGMA	Poly(glycidyl methacrylate)
PVA	Poly(vinyl alcohol)
R	Removal percentage
Rh	Hydrodynamic radius
SEM	Scanning electron microscopy
TGA	Thermogravimetric analysis
T_pol_	Polymerization temperature
UV-vis	Ultraviolet-visible spectroscopy
V	Volume of NaOH consumed in the sample (Equation (2))
$V_{0}$	Volume of NaOH consumed in the blank (Equation (2))
W	Mass of the sample (Equation (2))
WAC	Water absorption capacity
